# Parents’ and guardians’ perceptions of sexual and reproductive health communication with youth: A qualitative study in Gurage Zone, Southern Ethiopia

**DOI:** 10.1371/journal.pgph.0006825

**Published:** 2026-07-13

**Authors:** Getachew Gebreselassie Nida, Sisinyana H. Khunou, David Mphuthi

**Affiliations:** Department of Health Studies, College of Human Sciences, University of South Africa, Pretoria, South Africa; Durban University of Technology, SOUTH AFRICA

## Abstract

Youth sexual and reproductive health (YSRH) remains a significant global public health concern. Effective communication between parents and youth plays an important role in shaping young people’s knowledge, attitudes, and behaviors related to sexual and reproductive health (SRH). However, sociocultural norms and limited parental knowledge often hinder open discussions about SRH within families in many low- and middle-income countries, including Ethiopia. This study explored parents’ and guardians’ perceptions of SRH communication with youth in Gurage Zone, Southern Ethiopia. A qualitative study design was employed using focus group discussions (FGDs) with parents and guardians. Twelve participants aged 40–68 years participated in the discussions. Data were collected using a semi-structured interview guide exploring parental perceptions, attitudes, barriers and strategies related to SRH communication with youth. Audio recordings were transcribed, translated into English and analyzed using thematic analysis. The reporting of this qualitative study was guided by the Consolidated Criteria for Reporting Qualitative Research (COREQ) to enhance transparency, completeness and methodological rigor. Four major themes emerged: parental perceptions of SRH communication with youth; parental attitudes and beliefs regarding SRH communication; barriers to effective communication; and strategies to strengthen communication. Parents emphasized the importance of open dialogue, emotional safety and early discussions with youth. However, communication was often hindered by sociocultural taboos, generational gaps, limited parental knowledge and competing socioeconomic responsibilities. Parent–youth communication regarding SRH remains constrained by cultural norms and structural barriers. Strengthening parental knowledge and communication skills, alongside community-based interventions and multisectoral collaboration, may enhance youth access to accurate SRH information and promote healthier behaviors.

## Background

Youth sexual and reproductive health (YSRH) remains a major global public health and development priority. Youth aged 15–24 represent a large and growing population group whose health and well-being significantly influence social and economic development outcomes. Globally, youth face multiple sexual and reproductive health (SRH) challenges including early sexual initiation, unintended pregnancy, sexually transmitted infections (STIs) and HIV infection [1]. Limited access to accurate SRH information and supportive communication environments further increases youth vulnerability to risky sexual behaviors. Strengthening YSRH is also closely linked to the Sustainable Development Goals (SDGs), particularly SDG 3.7 which aims to ensure universal access to sexual and reproductive health services including family planning and reproductive health education by 2030 [2]. Promoting effective communication between youth and parents and guardians is increasingly recognized as a key strategy for improving YSRH outcomes.

From a global and international perspective, parents and guardians are important sources of socialization and guidance for youth regarding sexuality, relationships and reproductive health. Parent–youth communication on SRH has been shown to positively influence youth knowledge, attitudes and protective behaviors related to sexual health [3]. Evidence indicates that youth who communicate openly with parents about SRH issues are more likely to delay sexual initiation, adopt safer sexual practices and utilize reproductive health services [4]. Despite these benefits, discussions about sexuality within families remain limited in many cultural contexts due to taboos, embarrassment and concerns that discussing sexual matters may encourage early sexual activity [5]. As a result, many youths rely on peers, media or other informal sources for SRH information, which may be inaccurate or incomplete.

In Sub-Saharan Africa (SSA), youth face a disproportionate burden of sexual and reproductive health challenges compared to other regions. The region continues to report high levels of youth pregnancy, early marriage and HIV infection among youth people [6]. Although parents are widely recognized as influential actors in youth development, communication about SRH between parents and youth remains limited in many SSA communities due to socio-cultural norms that discourage open discussions about sexuality [7]. Studies across several African countries have shown that both parents and youth often perceive SRH discussions as uncomfortable or inappropriate, which reduces opportunities for youth to receive accurate information and guidance within the family setting [8]. Strengthening parent–youth communication has therefore become an important strategy in regional initiatives aimed at improving youth SRH outcomes.

In Ethiopia, youth represent a substantial proportion of the population and face several sexual and reproductive health challenges including early sexual initiation, unintended pregnancy and limited access to reliable SRH information [9]. Although the Ethiopian government has introduced youth-friendly health services and national strategies aimed at improving youth SRH, family-based communication on sexual and reproductive health issues remains limited. Studies conducted in different parts of Ethiopia indicate that cultural taboos, religious beliefs, parental discomfort, and limited knowledge about SRH topics contribute to low levels of parent–youth communication [10]. Evidence suggests that less than half of youth in Ethiopia report discussing SRH issues with their parents, highlighting persistent gaps in family communication on SRH matters [11].

Parents and guardians play a critical role in enhancing youth sexual and reproductive health by providing guidance, emotional support and accurate information regarding sexual health and relationships. When parents establish open and supportive communication with youth, they can help youth develop positive attitudes toward sexual health, strengthen decision-making skills and reduce engagement in risky sexual behaviors [6]. Parental engagement also facilitates trust and encourages youth to seek advice when facing sexual health concerns. Strengthening parents’ knowledge and communication skills through community-based interventions and health education programs may therefore contribute significantly to improving youth SRH outcomes.

Despite increasing recognition of the importance of parent–youth communication in promoting youth sexual and reproductive health, limited evidence exists on how parents and guardians perceive and approach discussions about SRH with youth in many Ethiopian communities. Cultural norms, social expectations and lack of parental confidence in discussing sexual health issues continue to hinder effective communication within families. Consequently, youth may lack reliable information and guidance regarding SRH [10]. Understanding parents’ and guardians’ perceptions of SRH communication with youth is therefore essential for designing culturally appropriate interventions that strengthen family communication and improve YSRH outcomes in Ethiopia, particularly in settings such as the Gurage Zone where empirical evidence remains limited.

## Methods and materials

### Study design

This study employed a qualitative research design using focus group discussions (FGDs) to explore parents’ and guardians’ perceptions regarding sexual and reproductive health communication with youth. The reporting was guided by the Consolidated Criteria for Reporting Qualitative Research (COREQ) to enhance transparency, completeness and methodological rigor. Qualitative methods were selected because it allow in-depth exploration of participants’ experiences, attitudes and social contexts related to sensitive topics such as sexuality and reproductive health. The research team consisted of trained researchers with experience in qualitative research and SRH studies. Prior to data collection, researchers received training on qualitative interviewing techniques and ethical considerations when discussing sensitive topics. To enhance reflexivity, the research team engaged in debriefing discussions after each FGD to reflect on the interview process, participant responses, emerging impressions and potential researcher assumptions. Field notes and reflective memos were used to document contextual observations and possible influences of the researchers’ professional backgrounds on data interpretation. During analysis, the team repeatedly discussed coding decisions to ensure that interpretations remained grounded in participants’ narratives rather than researchers’ prior assumptions

### Study setting

**Fig 1:**
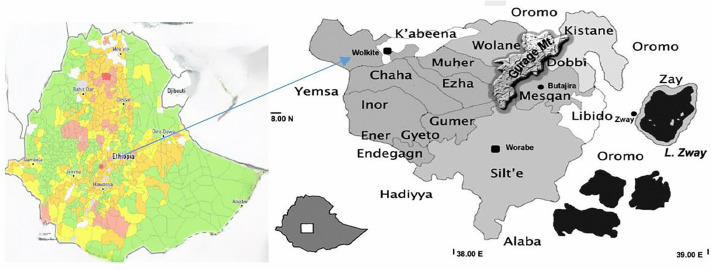
Administrative map of Gurage zone including districts/Woredas. (https://doi.org/10.1371/journal.pone.0198353.g001).

The study was conducted in Gurage Zone, Southern Ethiopia as part of a broader doctoral research examining parent–youth communication on SRH (Fig 1). Gurage Zone was purposively selected because of its sociocultural diversity and strong cultural and religious traditions, which are likely to influence family-level communication about sexuality, relationships and reproductive health.

### Participant recruitment

Participants were recruited using purposive sampling to ensure representation of parents and guardians with diverse backgrounds. Inclusion criteria included being a parent or guardian of youth and should have been living together for at least six months under the same roof during the time of the study. Eligible participants were adult parents or guardians who had parental or caregiving responsibility for youth.

### Data collection

Data were collected through focus group discussions using a semi-structured interview guide on April to May, 2025. Focus group discussions were selected because the study sought to explore shared parental perceptions, collective norms and culturally embedded beliefs regarding SRH communication with youth. FGDs were considered particularly appropriate because interaction among participants can stimulate discussion, reveal consensus and differences and generate rich data on socially sensitive issues shaped by community expectations. Individual interviews may have provided more private accounts; however, FGDs were better suited to capture collective meanings and normative views around parent–youth SRH communication in the study context

The semi-structured FGD guide was developed based on the study objectives, existing literature on parent–youth SRH communication and the broader doctoral research framework. The guide included questions on parental perceptions, attitudes, barriers, communication practices and strategies for strengthening SRH dialogue. It was reviewed by the supervisory research team for content relevance, cultural sensitivity and alignment with the study objectives. The guide was pretested with participants who had similar characteristics to the study population but were not included in the final FGDs. Feedback from the pretest was used to refine wording, sequencing and probing questions. Discussions were conducted in the local language and audio-recorded with participants’ consent. Each discussion lasted approximately 60–90 minutes.

Data saturation was monitored during data collection and preliminary analysis. After each FGD, the research team reviewed emerging issues, recurring codes and new insights. Saturation was considered achieved when subsequent discussions no longer generated substantially new themes but rather confirmed already identified categories including open communication, cultural taboos, gendered communication patterns, parental knowledge gaps, competing responsibilities and multisectoral strategies for improving SRH communication

### Data analysis and trustworthiness

Audio recordings from the interviews were transcribed verbatim and translated into English prior to analysis. The data were analyzed using thematic analysis guided by the framework proposed by Braun and Clarke (2006). Four researchers were involved in the data analysis process. The first author conducted the initial coding while the supervisory team reviewed the codes, emerging categories and themes. One independent coder also engaged with the analysis. The research team also brought complementary perspectives from public health, qualitative research and sexual and reproductive health. These varied disciplinary and experiential perspectives supported deeper interpretation of the data and helped strengthen reflexive engagement during analysis. The analysis began with familiarization with the data through repeated reading of the transcripts. This was followed by the generation of initial codes that captured meaningful segments of the data. The codes were then organized into potential themes which were subsequently reviewed and refined to ensure coherence and consistency with the dataset. Differences in coding and interpretation were resolved through iterative discussion and consensus meetings among the research team. Where interpretations differed, the team returned to the transcripts and participant quotations to ensure that final themes accurately reflected the data. Finally, themes were clearly defined and named to reflect their underlying meaning. To ensure the trustworthiness of the study, several strategies were implemented based on established qualitative research criteria. Credibility was enhanced through the involvement of multiple researchers who independently reviewed transcripts and coding decisions. Dependability was ensured by maintaining consistent coding procedures throughout the analysis process. Confirmability was achieved by grounding interpretations in participants’ narratives and supporting findings with direct quotations. Transferability was facilitated by providing detailed descriptions of the study context and participants, enabling readers to assess the applicability of the findings to other settings.

### Ethical considerations

Ethical clearance was obtained from the departmental higher degree research and ethics committee of University of South Africa (UNISA). A letter of permission from Gurage zone health department was granted to undertake the research providing Parents’ and Guardians’ Perceptions of Sexual and Reproductive Health Communication with Youth in the study area. The respondents were informed about the objective and purpose of the study. Oral and written consent was obtained from each respondent before the FGD conducted. To assure confidentiality no name or personal identifying information was written on the questionnaire and information was recorded anonymously

## Results

### Participants’ demographic information

Two focus Group discussion (FGD) involved 12 participants (6 females) aged between 40 and 68 years. Most participants were Orthodox Christians and married, though all four widowed individuals were women. Educational levels varied, with women generally having lower education compared to men. Employment also differed by gender; most women were housewives or engaged in informal work, while men held roles in agriculture, teaching, business, and civil service. The group reflects diverse socio-economic backgrounds with notable gender disparities (Table 1).

**Table 1:** Sociodemographic data for FGD participants.

Participants ID	Age	Sex	Educational Status	Religion	Marital Status	Employment
1	40	F	Grade 4	Orthodox	Widowed	Daily labourer
2	43	F	Not educated	Orthodox	Widowed	Housewife
3	49	F	Grade 3	Orthodox	Married	Merchant
4	51	F	Can read and write	Orthodox	Widowed	Housewife
5	52	F	Grade 3	Orthodox	Married	Housewife
6	40	F	Grade 10	Orthodox	Married	Housewife
7	58	M	Grade 7	Orthodox	Married	Merchant
8	56	M	Degree	Orthodox	Married	Civil servant
9	63	M	Diploma	Protestant	Married	Agriculture
10	68	M	Grade 10	Orthodox	Married	Agriculture
11	44	M	Diploma	Orthodox	Married	Merchant
12	62	M	Diploma	Orthodox	Married	Teacher

## Emergent themes and sub-themes

This study explored and described Parents’ and Guardians’ Perceptions of Sexual and Reproductive Health Communication with Youth. Analysis of the data resulted in the identification of four major themes reflecting Parents’ and Guardians’ Perceptions of Sexual and Reproductive Health Communication with Youth at different levels. These themes emerged through a systematic process of coding and categorizing participants’ responses to questions related to their perceptions of SRH communication. Illustrative phrases and direct quotations corresponding to the identified codes are presented under each emergent theme and sub-theme (see Table 2).

**Table 2:** Themes, categories and sub-categories that emerged from Focus Group Discussion.

Themes	Sub-themes	Categories
1. Parental perception on the SRH communication with youth	1.1. Parental roles and approaches	1.1.1. Open communication
		1.1.2. Avoiding authoritarianism
		1.1.3. Emotional safety
		1.1.4. Parental engagement
	1.2. Timing and initiation of discussions	1.2.1. Early start (Ages 9–12)
		1.2.2. Age-appropriate topics
2. Parental attitudes and beliefs on SRH communication	2.1. Parental attitudes on SRH communication	2.1.1. Perceived importance of SRH communication
		2.1.2. Parental attitudes
		2.1.3. Gendered perspectives
		2.1.4. Intergenerational learning and role modeling
3. Barriers to effective SRH communication	3.1. Sociocultural and generational challenges	3.1.1. Secrecy and shame
		3.1.2. Generational resistance
	3.2. Parental knowledge and attitude gaps	3.2.1. Parental illiteracy/Lack of skills
		3.2.2. Competing priorities
		3.2.3. Health consequence of using tradition medicine
4. Strategies and enablers for effective communication	4.1. Collaboration among different stakeholders and roles	4.1.1. Government and non-governmental organisation active participation
		4.1.2. School and religious roles
		4.1.3. Community involvement and engagement
	4.2. Practical parental approaches on SRH communication	4.2.1. Creating safe spaces
		4.2.2. Media management and modelling
		4.2.3. Encourage open conversation with youth
	4.3 Health education and awareness creation on SRH	4.3.1. Effective educational roles of the parents
		4.3.2. Peer education on SRH

### Theme 1: Parental perception on the SRH communication with youth

Effective communication between parents and youth regarding SRH is crucial for promoting healthy behaviors and informed decision-making among youth. However, various factors influence the effectiveness of this communication, including parental perceptions, communication styles, and societal norms.

#### Sub-them 1.1: Parental roles and approaches.

Participants agreed that open communication is key to effective youth SRH education and service uptake. They emphasized that when youth can speak freely with parents without fear of judgment, it builds trust, dispels myths, and encourages informed decision-making and timely care-seeking behavior. Parents emphasized:

*“… we need to foster openness within our youth. It’s crucial that children understand they have a role and that their education and personal development are priorities. My message is clear: let’s not be secretive. Open communication is vital for preventing issues and guiding our youth effectively”* ***(P8).***

Parents reported the need to avoid a controlling or harsh approach when discussing SRH topics with youth, as it leads to fear and disengagement. This is supported by this expression:

*“Many parents struggle with dynamics of power in their relationships, often treating one another unequally. This secrecy persists; they may live in isolation, and their youth often follow suit. Fathers may use threats to control their youth, which creates further obstacles”* ***(P11****).*

#### Sub-theme 1.2: Timing and initiation of discussions.

Parents emphasized that the timing and initiation of SRH discussions are critical. They noted that starting conversations early and discussing age- appropriate topics. Most parents believed early initiation of SRH discussions helps normalize the topic and still protective behaviors before youth are exposed to misinformation. This is supported by the following quotation:

*“In the past, we often started teaching this around 15 years old, but now I see that girls as young as 12 and 13 are facing serious issues. I believe we should begin educating them as early as 9 or 10. It’s beneficial to provide this knowledge early since younger children tend to learn more quickly and easily”* ***(P6).***

Parents highlighted the need for delivering age-appropriate SRH topics to effectively educate and engage youth. They emphasised that tailoring information to the developmental stage and understanding level of young people helps ensure relevance, fosters trust, and encourages healthy attitudes and behaviours without overwhelming or misinforming them. Parents highlighted:

*“We cover a wide range of subjects. I talk to them about various issues and remind them to avoid the wrong places and relationships. I tell my daughter, “Now that you’re old enough for a relationship, be careful not to get involved with the wrong friends and peers”* ***(P1).***

### Theme 2: Parental attitudes and beliefs on SRH communication

Parental attitudes greatly influence SRH communication with youth. This qualitative focus group discussion analysis revealed four major sub-categories: 1) Perceived importance of SRH communication, 2) Parental attitudes, 3) Gendered perspectives and 4) Intergenerational Learning and role modelling.

#### Sub-them 2.1: Perceived importance of SRH communication.

Many parents acknowledged that communication on SRH matters is essential in preventing risky sexual behaviours and promoting healthy development in youth. This is demonstrated by this quotation:

*“My own upbringing plays a role. I didn’t receive much education on these issues when I was young, and I see the consequences of that in my community. I want to ensure my children are better informed and prepared to make safe choices. These are the main reasons that drive me to have these important discussions with my children”* ***(P3).***

Participants indicated that parental attitudes play a significant role in shaping youth access to SRH information. While some parents are supportive, many hold conservative or hesitant views due to cultural norms, discomfort, or lack of knowledge. This is supported by this quotation:

*“If youth are engaged in productive activities, they’re less likely to fall into harmful behaviours. The primary responsibility lies with the family; success comes from nurturing youth with love. Open communication can help mitigate negative behaviours”* ***(P9).***

#### Sub- theme 2.2: Parental attitudes.

Participants indicated that parental attitudes play a significant role in shaping youth access to SRH information. While some parents are supportive, many hold conservative or hesitant views due to cultural norms, discomfort, or lack of knowledge. This is supported by this quotation:

*“We saw that they could have a better life if we included them in our family. Thankfully, they are now living with us, and it’s been beneficial for everyone. I believe it’s important to spend time with our youth and listen to them”* ***(P5).***

In agreement with the discussant:

*“If youth are engaged in productive activities, they’re less likely to fall into harmful behaviours. The primary responsibility lies with the family; success comes from nurturing youth with love. Open communication can help mitigate negative behaviours”* ***(P9).***

Evidence consistently shows that parent–youth communication on SRH remains limited, shaped by cultural norms, parental attitudes, and socio-economic context. Usonwu et al., (2021:8) established that cultural and religious restrictions, coupled with parental discomfort and lack of knowledge, often reduce conversations to silence or fear-driven warnings.

#### Sub- theme 2.3: Gendered perspectives.

Gender dynamics shaped how SRH discussions were approached. Fathers were often less involved, and topics differed depending on the child’s gender. Mothers tended to discuss menstruation and relationships with daughters. This is demonstrated by this quotation:

*“Many young women spend significant time with their mothers and, although they love their fathers, they tend to confide in their mothers more openly. Mothers are typically more straightforward; when a secret is shared, they keep it private. I think it would be beneficial to focus on mothers to encourage openness between them and their daughters. This approach fosters a deeper connection and understanding, which is often more accepted and intimate”* ***(P10).***

Another participant added:

*“We need to be aware of their arrival and departure times from school, treating both boys and girls equally but paying extra attention to our daughters since they may be more vulnerable. If my daughter is even a couple of minutes late, I’ll go check on her to ensure she’s safe. I talk to her about what she might encounter from boys and advise her on how to respond, using real-life examples to illustrate the importance of my guidance”* ***(P6).***

In agreement with the other participants:

*“Girls are more at risk, so I talk to them more often than I do with my sons. Mothers share a deep connection with daughters... They keep secrets private”* ***(P11*)**.

Gender norms profoundly shape parent–youth SRH communication across different contexts that cultural taboos around sexuality perpetuate a culture of silence, particularly silencing girls and limiting their ability to express SRH concerns openly.

#### Sub- theme 2.4: Intergenerational learning and role modelling.

Parents’ own experiences positive or negative with SRH communication during their youth influence how they engage with their youth. One of the participants had this to say:

*“I have four daughters, all of whom are married, but one experienced rape and became pregnant. I decided to stop that marriage, despite her feelings for him. She was pregnant at the time, and now her daughter is 14. I am teaching her about these issues, using her mother’s experience as a lesson. I go beyond just discussing menstruation; I share what I’ve learned from my own experiences”* ***(P4).***

This is also supported by this quotation:

*“I stress the importance of honesty between parents and children, sharing my own experiences with the harms of alcohol to provide context. I believe in the importance of timing, there’s a right time for everything, including marriage and celebrations. Knowledge gained at home is as valuable as formal education, and I teach my children how to navigate economic challenges”* ***(P9).***

Evidence shows that intergenerational storytelling and dialogue play an important role in strengthening parent–youth communication and supporting youth SRH outcomes

### Theme 3: Barriers to effective SRH Communication

Parents identified several barriers to effective SRH communication with youth. Major categories emanating from the FGD analysis are: 1) Sociocultural and generational challenges and 2) Parental knowledge and attitude gaps. This theme reflects deep-rooted cultural norms and intergenerational tensions that suppress open dialogue about sexuality and reproductive matters within families.

#### Sub-theme 3.1: Sociocultural and generational challenges.

Parents highlighted that secrecy and shame are major obstacles to effective SRH communication among youth. Cultural taboos and fear of judgment often prevent open discussions, leading young people to seek information from unreliable sources or avoid services altogether. This is supported by this expression:

*“Secrecy often stems from a parent who acts like a head of the household without engaging with the family. They come and go without involving their children in discussions. This approach leads to a disconnect between their views and those of their children”* ***(P7).***

Parents also noted that generational resistance is a key barrier to youth SRH communication and service uptake. This is supported by this quotation:

*“One major challenge is the lack of willingness among the youth to listen and engage. As my brother mentioned, some are resistant to accepting advice, saying things like, you had your fun in your youth; if we face issues now, we’ll handle it our own way. We’re also seeing troubling trends, such as young women abandoning their babies”* ***(P12).***

#### Sub-them 3.2: Parental knowledge and attitude gaps.

Low literacy levels among parents significantly affect their ability to provide accurate SRH information. Many lacks the knowledge, confidence, or communication skills required to discuss sensitive topics effectively. One of the participants had this to say:

*“…Additionally, my own upbringing plays a role. I didn’t receive much education on these issues when I was young, and I see the consequences of that in my community. I want to ensure my children are better informed and prepared to make safe choices”* ***(P3****).*

Parents noted that competing priorities, such as economic pressures, household responsibilities, and work demands, often limit parents’ ability to focus on youth SRH. This is demonstrated by this quotation:

*“Mothers are busy working... we can’t rely solely on the government”* ***(P3).***

### Theme 4: Strategies and enablers for effective communication

This theme captures the strategies and enabling conditions that facilitate improved communication between parents and youth about SRH. Insights emerged from participants emphasising [1] the roles of multisectoral collaboration, [2] parental strategies, and [3] targeted health education to overcome communication barriers and promote informed youth decision-making.

#### Sub-theme 4.1: Collaboration among different stakeholders and roles.

Active involvement of government ministries and NGOs is crucial for advancing SRH communication initiatives. These stakeholders play a vital role in policy development, technical assistance, and community-based programming. This is supported by this expression:

*“This is where government intervention becomes critical. Awareness initiatives need to continue extensively. The community also has a responsibility; the consequences of these SRH issues affect everyone. We cannot just focus on the youth; we need to engage with them based on their behaviour and support them as necessary”* ***(P12).***

Schools and religious areas serve as strategic platforms for disseminating age-appropriate SRH information and fostering dialogue between students and their families. One of the participants reported:

*“I learnt that an HIV-related curriculum has been adopted, and students are being taught this as a subject, especially alongside road safety. This education is crucial and timely for our country, starting from Grade 1. While we know that 7-year-olds aren’t involved in sexual activities, it’s important to educate them early about the risks associated with SRH and HIV/AIDS and its impacts as they grow older”* ***(P8).***

Community-based initiatives, such as discussion forums and youth-adult dialogues, create opportunities for safe engagement on SRH topics. This is demonstrated in this submission:

*“Awareness-raising activities are crucial but are not happening at the community level. There’s a need for activists who focus on grassroots efforts. Unfortunately, many people are preoccupied with their own issues and neglect to support others. This gap is evident, and the government is also failing to provide essential awareness initiatives. Everything should begin at the community level and then move upward. however, this approach is not being implemented”* ***(P10).***

#### Sub-theme 4.2. Practical Parental Approaches on SRH communication.

Participants suggested that parents need both practical tools and a supportive environment to communicate effectively with their youth. A safe, non-judgmental space for open dialogue was seen as essential for encouraging youth to ask questions and express themselves regarding SRH. This is supported by this expression:

*“Teaching good values benefits not only the individual but also contributes to the development of the country. We can’t rely solely on teachers or others; we must take responsibility for the youth around us. By guiding our neighbours’ children and instilling good manners, we help them become responsible citizens. If we all adopt this approach, I believe we can collectively foster positive change in our country”* ***(P11).***

Parents’ regulation of media exposure and their modelling of respectful, informed behaviour plays a critical role in shaping youth attitudes toward SRH.

One of the participants had this to say:

*“Our youth are influenced by what they see on their phones. They beg us for smartphones, but they end up watching harmful content. We often don’t even watch the news because it’s painful to see. Even when we try to restrict their TV time, they find ways to watch elsewhere or on their phones, exposing themselves to inappropriate content related to SRH that fosters cruelty and immorality” (* ***P3).***

Parents emphasised the importance of encouraging open conversations with youth about SRH. They noted that creating safe, respectful spaces for dialogue builds trust, empowers young people with accurate information, and helps them make informed decisions. This is supported by this expression:

*“Open dialogue is crucial; it not only helps the youth but also protects their families from potential problems. Personally, I believe it’s very useful to talk about these matters. Keeping things hidden can lead to surprises down the line. I think it’s important to discuss both the merits and demerits freely”* ***(P2).***

#### Sub-theme 4.3: Health education and awareness creation on SRH.

Participants emphasised that parents play a crucial educational role in shaping their children’s understanding of SRH. This is demonstrated in this quotation:

*“I believe it’s essential for children to be educated on these topics, especially by their mothers, from ages 14 to 25. Ignorance, particularly among those who are illiterate, can be dangerous”* ***(P4).***

Parents recognised peer education as a highly effective strategy for promoting SRH among youth. This is supported by this expression:

*“I tell him that bad friends can lead to trouble. I’ve seen his friends engaging in sexual activities in inappropriate places. I warn him that having sex can lead to diseases, and that addiction can cause poor academic performance and a lack of direction in life”* ***(P2).***

## Discussion

This study explored parents’ and guardians’ perceptions of SRH communication with youth in Gurage Zone, Southern Ethiopia. The findings reveal that while parents recognize the importance of discussing SRH issues with youth, multiple sociocultural, structural, and generational barriers limit effective communication.

The study found that open communication and emotional safety within families were perceived as critical facilitators of effective SRH dialogue. Parents emphasized that youth are more likely to seek guidance and share concerns when communication occurs in supportive and non-judgmental environments. This finding is consistent with previous research demonstrating that open parent–youth communication improves youth knowledge and promotes safer sexual behaviors [12]. Studies conducted in both high-income and low-income settings have shown that youth who communicate regularly with parents about SRH are more likely to delay sexual initiation and adopt protective behaviors [3,13]. The findings suggest a critical tension between parental recognition of the importance of SRH communication and the sociocultural constraints that limit its practice. Although participants acknowledged that open dialogue could protect youth from misinformation, unintended pregnancy, STIs and unsafe practices, communication remained constrained by shame, secrecy, gender norms and limited parental confidence. This indicates that improving parent–youth SRH communication requires more than encouraging parents to talk, it requires addressing the cultural, relational and structural conditions that determine whether such conversations are possible, acceptable and effective

Participants also emphasized the importance of initiating SRH discussions during early adolescence. Early communication helps normalize conversations about sexuality and equips youth with knowledge before they encounter misinformation from peers or media. Previous studies have similarly demonstrated that early sexuality education improves youth knowledge and reduces risk behaviors [14–16]. Interventions targeting early youth have been shown to significantly improve reproductive health outcomes by promoting healthy attitudes and informed decision-making [1].

Despite recognizing the importance of communication, parents in this study reported that cultural taboos surrounding YSRH remain a major barrier. Similar findings have been reported in multiple studies across Sub-Saharan Africa, where discussions about SRH within families are often restricted by social norms and stigma [5]. These cultural constraints contribute to a culture of silence that limits youth access to reliable information and increases reliance on peers or digital media [17,18]. The results confirm previous evidence from Ethiopia and Sub-Saharan Africa showing that parent–youth SRH communication is constrained by cultural taboos, embarrassment, limited parental knowledge and gendered expectations. However, this study extends existing evidence by demonstrating that parents and guardians are not merely barriers to SRH communication, they also identify contextually acceptable strategies for change including early age-appropriate discussion, non-authoritarian parenting, emotional safety school and religious engagement, peer education, community dialogue and multisectoral support

Gender norms also influenced communication patterns in this study. Mothers were more likely to engage daughters in SRH discussions, while fathers were less involved. Similar gendered communication patterns have been observed in several African contexts [19,20]. Such patterns may reinforce gender inequalities in access to sexual health information.

Another important finding relates to parental knowledge and educational status. Participants indicated that limited knowledge and lack of confidence hinder their ability to discuss SRH issues effectively. Previous research has shown that parents with higher levels of education and SRH knowledge are more likely to communicate with youth about sexuality [4,21]. Interventions that improve parental knowledge and communication skills have been shown to significantly strengthen parent–child dialogue and improve youth health outcomes [4,17].

Participants also highlighted economic pressures and competing responsibilities as barriers to communication. Parents reported that work obligations and household responsibilities often limit opportunities for discussions with youth. Similar findings have been reported in other studies examining family communication in resource-limited settings [18,22].

Importantly, participants emphasized the potential role of community-based and multisectoral interventions in improving SRH communication. Collaboration between families, schools, community organizations, and health institutions was identified as an important strategy. Evidence from previous research suggests that integrated approaches involving families and community institutions are more effective in improving youth sexual and reproductive health outcomes [1,17]. The strategies identified by participants suggest that interventions should operate at multiple levels. At the family level, parents need practical communication skills including how to initiate age-appropriate conversations, listen without judgment, respond to youth questions and discuss sensitive topics such as puberty, relationships, pregnancy, STIs/HIV, contraception and unsafe abortion. At the community level, dialogue forums involving parents, youth, religious leaders, schools, health workers and community organizations may help reduce shame and normalize SRH communication. At the institutional level, schools and health facilities can support parents by providing accurate SRH information, referral pathways and structured family engagement sessions. These findings suggest that parent-focused SRH communication interventions should be integrated into community health, school health, and youth-friendly service program.

The practical implications include the need for parent-focused SRH communication training delivered through health extension workers, schools, religious institutions and community-based organizations. Health workers can provide parents with accurate SRH information and communication tools. Schools can create structured parent–student dialogue opportunities. Religious and community leaders can help reduce stigma by framing SRH communication as a protective family responsibility rather than a moral threat. NGOs and local health offices can support community awareness campaigns, peer education and youth-friendly referral systems. Such interventions should be culturally sensitive, gender-responsive and adapted to parents’ literacy levels.

Overall, the findings of this study highlight the need for interventions that strengthen parental communication skills, address sociocultural barriers, and promote supportive family environments for discussions about sexual and reproductive health.

## Conclusion

This study provides context-specific insights into parents’ and guardians’ perceptions of SRH communication with youth in Gurage Zone, Southern Ethiopia. Parents recognized the importance of open, early and supportive communication, but sociocultural taboos, gender norms, limited parental knowledge, generational differences and competing socioeconomic responsibilities constrained effective dialogue. Strengthening parent–youth SRH communication requires culturally sensitive, family-centered and multisectoral interventions involving parents, youth, schools, health workers, religious leaders, community structures and NGOs.

## Supporting information

S1 Checklist Checklist. (DOC)
