# Supplementary material for: Parents’ and guardians’ perceptions of sexual and reproductive health communication with youth: A qualitative study in Gurage Zone, Southern Ethiopia
Source: PLOS Glob Public Health. 2026 Jul 13;6(7):e0006825. doi: 10.1371/journal.pgph.0006825 (PMC13362135; doi:10.1371/journal.pgph.0006825)
Supplement: S1 Checklist — (DOC) [file pgph.0006825.s001.DOC]

Inclusivity in global research

PLOS’ policy on inclusivity in global research aims to improve transparency in the reporting of research performed outside of researchers’ own country or community and ensures that PLOS publications reporting global research adhere to high standards for research ethics and authorship. Authors of relevant research articles may be asked to complete the questionnaire below, which outlines ethical, cultural, and scientific considerations specific to inclusivity in global research. This questionnaire may be requested when researchers have travelled to a different country to conduct research, if research uses samples collected in another country, research with Indigenous populations or their lands, or if research is on cultural artefacts. Researchers travelling to another country solely to use laboratory equipment will not normally be required to complete the questionnaire. However, the questionnaire can be requested at the journal’s discretion for any submission – if you have been requested to complete this questionnaire by the PLOS journal you submitted to, please do so.

Please complete the questionnaire below and include this as a Supporting Information file with your manuscript. Note that if your paper is accepted for publication, this checklist will be published with your article in the supporting information files. Please ensure that you reference the checklist in the main body of your manuscript. We suggest adding a subsection ‘Inclusivity in global research’ to your Methods section and adding the following sentence: “Additional information regarding the ethical, cultural, and scientific considerations specific to inclusivity in global research is included in the Supporting Information (SX Checklist)”

The questions have been designed to be applicable to a wide range of study types, and there are subsections for both human subjects research and non-human subjects research. If any of the questions are not relevant to your research please mark them as “N/A” as appropriate.

**Ethical considerations, permits and authorship**

*This section is applicable to all research types.*

Provide details as to who granted permissions and/or consent for the study to take place in the Methods section of your manuscript. This should include the names of **all** ethics boards, governmental organizations, community leaders or other bodies that provided approval for the study. If individuals provided approval refer to these people by their role or title but do not list their name(s).

Ethical approval for this study was obtained from the Department of Health Studies Higher Degrees Committee and the Research Ethics Committee of the University of South Africa (UNISA). Administrative permission to conduct the study was obtained from the Gurage Zone Health Department and relevant local administrative authorities prior to data collection.

Community-level entry and coordination were facilitated through local health and administrative structures. Eligible participants were informed about the purpose, objectives, procedures, benefits, and potential risks of the study before participation. Participants were also informed that their participation was voluntary and that they had the right to decline participation or withdraw from the study at any stage without any negative consequences.

Written informed consent was obtained from all participants before the focus group discussions were conducted. To maintain confidentiality and privacy, no personal identifiers were recorded in the transcripts or reports, and audio recordings and transcripts were stored securely with access restricted to the research team only.

Because the study addressed sensitive issues related to sexual and reproductive health communication, efforts were made to create a respectful, non-judgmental, and culturally sensitive discussion environment during data collection. Page 8

If there were any deviations from the study protocol after approval was obtained please provide details of these changes in the Methods section of your manuscript.
Did this study involve local collaborators that are residents of the country where the research was conducted or members of the community studied? If you do not have any authors from said communities, please provide an explanation for this below.

NA. There is no any change

Reported on page number:

Everyone listed as an author should meet PLOS’ criteria for authorship and all individuals who meet these criteria should be included in the author byline, rather than the acknowledgements. For further information please see the journal’s Authorship Policy.

**Human subjects research (e.g. health research, medical research, cross-cultural psychology)**

Did you obtain written informed consent from a representative of the local community or region before the research took place? How did you establish who speaks for the community? Details of written informed consent obtained from study participants should be reported separately in the Methods section of your manuscript.

Before data collection commenced, official permission to conduct the study was obtained from the relevant institutional and local administrative authorities, including the Gurage Zone Health Department and local district-level administrative and health structures. Community entry procedures were conducted through local administrative and health officials who facilitated communication with the community and supported identification of eligible participants.

Because this study involved adult participants and did not involve community-level interventions or experimentation, formal proxy consent from a single individual speaking on behalf of the entire community was not considered appropriate. Instead, engagement with local authorities and community structures was used to ensure cultural appropriateness, local acceptance, and ethical conduct of the research within the study setting.

Written informed consent was obtained individually from all study participants prior to participation. Participants were informed about the purpose of the study, voluntary participation, confidentiality protections, and their right to withdraw at any stage without consequences. Details regarding individual informed consent procedures are provided in the Methods section of the manuscript.

How did members of the local community provide input on the aims of the research investigation, its methodology, and its anticipated outcome(s)?

Members of the local community contributed to the research process through engagement with local health and administrative structures during the planning and implementation phases of the study. Prior to data collection, discussions were held with local health officials and community-level stakeholders to ensure that the research topic, objectives, and procedures were contextually relevant and culturally appropriate within the Gurage Zone setting.

The study aimed to explore parents’ and guardians’ perceptions of sexual and reproductive health (SRH) communication with youth, an issue identified locally as socially sensitive and influenced by cultural and religious norms. Input from local stakeholders helped refine the focus of the investigation toward understanding community-specific barriers, communication practices, and culturally acceptable approaches for improving SRH dialogue between parents and youth.

Community perspectives also informed the methodological approach. The use of focus group discussions was considered appropriate because it enabled participants to discuss shared experiences, social norms, and culturally embedded perceptions in an interactive setting. In addition, pilot testing and feedback from individuals familiar with the local context helped improve the clarity, cultural sensitivity, and appropriateness of the discussion guide and terminology used during data collection.

The anticipated outcomes and practical recommendations of the study were also shaped by participants’ contributions. Community members emphasized the importance of culturally sensitive communication strategies, family engagement, school involvement, youth-friendly health services, community awareness creation, and multisectoral collaboration. These perspectives informed both the interpretation of findings and the recommendations presented in the manuscrip

When engaging with the local community, how did you ensure that the informed consent documents and other materials could be understood by local stakeholders?

To ensure that informed consent documents and study materials were understandable and culturally appropriate for local stakeholders, all participant information sheets, consent forms, and discussion guides were prepared in the local language commonly spoken in the study area. The materials were translated from English into the local language using terminology that was simple, culturally sensitive, and appropriate to the literacy level of participants.

Before data collection, the research team reviewed the translated materials to ensure conceptual consistency, clarity, and cultural appropriateness. The discussion guide was also pretested with individuals who had similar characteristics to the study participants but were not included in the final study. Feedback from the pretest helped refine wording, improve clarity, and ensure that sensitive sexual and reproductive health terminology was understandable and respectful within the local cultural context.

During recruitment and prior to participation, the consent information was explained verbally by trained data collectors in the local language to ensure that participants fully understood the purpose of the study, procedures, voluntary nature of participation, confidentiality protections, potential risks and benefits, and their right to withdraw at any time without consequences. Participants were also given opportunities to ask questions and seek clarification before providing written informed consent.

Will the findings of the research be made available in an understandable format to stakeholders in the community where the study was conducted (e.g. via a presentation, summary report, copies of publications, etc.)? Please provide details of how this will be achieved.

Yes. The findings of the study will be shared with relevant stakeholders in the study area through locally appropriate and understandable dissemination approaches. The research team intends to provide summary feedback to local health authorities, including the Gurage Zone Health Department and relevant district-level health offices, which supported implementation of the study.

The findings will also be disseminated through presentations and discussions with local stakeholders, including health workers, community representatives, and other relevant actors involved in youth sexual and reproductive health programs. Efforts will be made to communicate the findings in clear and non-technical language to ensure accessibility and practical relevance for community stakeholders.

In addition, the published article and summary findings may be shared electronically with relevant institutions and stakeholders to support awareness, policy dialogue, and future program planning related to parent–youth sexual and reproductive health communication. The dissemination process will emphasize culturally appropriate communication and practical recommendations that are relevant to the local context.

**Non-human subjects research using specimens/ animals collected as part of the study, or those housed in archival collections. Examples include archaeology, paleontology, botany and zoology.**

Did the permission you obtained from a local authority to perform the study include an agreement on access to outputs and benefit sharing? This may include procedures to enable fair distribution of the benefits and resources arising from the research performed. Please include any details of Prior Informed Consent and Benefit Sharing Agreements obtained. These may be required by field-specific regulations, for example the Convention on Biological Diversity (CBD) and the associated Nagoya Protocol.

NA

If the material used in your study was imported, please A) provide the year it was imported and B) indicate whether permits were obtained to import/export the materials used, C) provide details of any permits obtained. If this information is not available, please indicate this.

NA

If you used archival specimens, please state how the material used in your study was acquired by the institute it is held in and provide details of any permits obtained for the original excavations/ sample collection. If this information is not available, please indicate this.

NA

How was the potential cultural significance of the materials collected in your study to local communities considered in your research design? Were Indigenous peoples and/or local researchers and institutions involved with archaeological excavations / collection of specimens? If so, please provide a description of their involvement.

NA

If your manuscript includes photographs of human remains please indicate whether authors obtained permission from descendants or affiliated cultural communities to do so.

NA
