# Supplementary material for: Parents’ and guardians’ perceptions of sexual and reproductive health communication with youth: A qualitative study in Gurage Zone, Southern Ethiopia
Source: PLOS Glob Public Health. 2026 Jul 13;6(7):e0006825. doi: 10.1371/journal.pgph.0006825 (PMC13362135; doi:10.1371/journal.pgph.0006825)
Supplement: Attachment — Submitted filename: Justication for the comment.pdf [file pgph.0006825.s004.pdf]

Comment:
Ethical approval was not obtained from an Ethiopian ethics committee. Please provide justification.

Response:
Thank you for this important comment. Ethical approval for this study was obtained from the University of South Africa (UNISA) Research Ethics Committee as part of the requirements for PhD fulfillment. In addition, official permission to conduct the study was obtained from the Gurage Zone Health Department in Ethiopia prior to data collection.

At the time of the study, separate ethical approval from a local Ethiopian Institutional Review Board was not sought because the study was conducted under the academic oversight of UNISA, and local administrative clearance was secured through the relevant health authority. The study involved minimal risk to participants, and all ethical principles, including voluntary participation, informed consent, confidentiality, and anonymity, were strictly maintained.
