# Supplementary material for: Parents’ and guardians’ perceptions of sexual and reproductive health communication with youth: A qualitative study in Gurage Zone, Southern Ethiopia
Source: PLOS Glob Public Health. 2026 Jul 13;6(7):e0006825. doi: 10.1371/journal.pgph.0006825 (PMC13362135; doi:10.1371/journal.pgph.0006825)
Supplement: Attachment — Submitted filename: Feedback_Reviewer.pdf [file pgph.0006825.s002.pdf]

Based on the provided manuscript, the Ethiopian study utilized a qualitative approach focused on a specific geographic and demographic subset. Below is an in-depth analysis of the sample size and its implications for the research findings.

Sample Size Overview:

Total Participants: The study consisted of 12 participants.
Composition: The group was balanced by gender, including 6 females and 6 males.
Age Range: Participants were aged between 40 and 68 years.
Geographic Focus: All participants were residents of the Gurage Zone in Southern Ethiopia.

Methodological Context of the Sample: The study employed purposive sampling. This non-probability sampling method was chosen to ensure representation from parents and guardians with diverse backgrounds, including variations in education (ranging from uneducated to degree holders) and employment (housewives, merchants, civil servants, and farmers).

Impact of Sample Size on Findings:

a. Depth of Exploration: Because the sample size was small (N=12), the researchers utilized Focus Group Discussions (FGDs) lasting 60–90 minutes to capture rich, descriptive data. This allowed for the emergence of nuanced themes regarding sociocultural taboos and "secrecy," which might be harder to quantify in a larger survey-based study.
b. Reaching "Saturation: "In qualitative research, the goal is often data saturation—the point where no new information or themes emerge from the data. The researchers identified four major themes (perceptions, attitudes/beliefs, barriers, and strategies) from this group, suggesting that despite the small number, the participants provided sufficient diversity in thought to establish a comprehensive framework for the study area.
c. Transferability vs. Generalizability: Limited Generalizability due to the small sample size and specific focus on the Gurage Zone mean the findings cannot be statistically generalized to the entire Ethiopian population. High Transferability observed due to the author's action to address this by providing "thick descriptions" of the context and participants. This allows readers to assess how the findings regarding "generational resistance" or "cultural taboos" might apply to other similar rural, tradition-heavy settings in Sub-Saharan Africa.
d. Impact on Sub-group Analysis: The small size (N=12) limits the ability to perform detailed comparisons between different demographic sub-groups within the sample. For example, while the study notes that all four widowed participants were women and that women generally had lower education levels than men, the sample is too small to definitively correlate these specific traits with unique SRH communication styles.
e. Mitigation of Bias: To counter the potential limitations of a small sample, the study implemented trustworthiness strategies, such as involving multiple researchers to review transcripts (credibility) and grounding interpretations strictly in the participants' direct narratives (confirmability)

Based on the study conducted in the **Gurage Zone, Southern Ethiopia**, the researchers provided several key recommendations aimed at overcoming cultural barriers and structural challenges to improve youth sexual and reproductive health (YSRH).

Implementation Strategies

These focus on practical, community-level actions to bridge the communication gap between parents and youth:

- **Enhancing Parental Knowledge and Skills:** Interventions should focus on improving parents' SRH knowledge and communication skills. This helps parents gain the confidence needed to discuss sensitive topics effectively.
- **Community-Based Awareness Programs:** Grassroots initiatives, such as discussion forums and youth-adult dialogues, should be implemented to create safe spaces for engagement.
- **Leveraging Strategic Platforms:** Schools and religious institutions should be utilized as platforms to disseminate age-appropriate SRH information and foster family dialogue.
- **Peer Education:** Integrating peer education is recommended as a highly effective strategy for promoting SRH among youth within the community.
- **Media Management Training:** Providing parents with strategies to manage their children's media and smartphone exposure to prevent the consumption of harmful or inappropriate content.

Policy Recommendations

These focus on broader systemic changes to support family-based SRH communication:

- **Multisectoral Collaboration:** The study advocates for strong collaboration between families, schools, health institutions, government ministries, and NGOs to advance SRH initiatives.
- **Early Education Policies:** Policies should support the initiation of SRH education during early adolescence (starting as early as age 9 or 10) to normalize the topic before youth are exposed to misinformation.
- **Supportive Educational Curricula:** Formal adoption and expansion of SRH and HIV-related curricula in schools, starting from early grades (e.g., Grade 1), to build a foundational understanding of risks and impacts.
- **Addressing Economic and Structural Barriers:** Policymakers need to recognize that competing socioeconomic responsibilities and economic pressures limit parental engagement, suggesting a need for broader support systems for families.
- **Integration of Services:** Enhancing the reach of youth-friendly health services by ensuring they are culturally appropriate and supported by community awareness programs.

Comparison to Malawi Study

The recommendations comparison to the specific "outside expert" and role model approach suggested in the Malawi study[1].

The comparison between the Ethiopian and Malawian recommendations reveals a shift in who is perceived as the most effective messenger for sexual and reproductive health (SRH) information. While the Ethiopia study focuses on strengthening existing community and family structures, the Malawi study emphasizes the need for external figures to break through local cultural constraints.

Messenger: Internal vs. ExternalEthiopia Study (Internal/Structural): The recommendations prioritize empowering parents, teachers, and religious leaders as the primary educators. The goal is to improve the "internal" communication skills of those already in the youth's immediate environment.
Malawi Study (External/Aspirational): There is a specific call for "outside experts" and role models from "far away". These figures are seen as having more credibility because they are not part of the daily household power dynamics where girls may become "rude and challenging" to their parents.

Objective: Guidance vs. Alternative VisionEthiopia Study (Guidance & Risk Management): The focus is on risk reduction and healthy decision-making. Parents and stakeholders are encouraged to create "safe spaces" to help youth avoid "bad friends," "harmful content" on smartphones, and "immorality".
Malawi Study (Aspiration & Future Paths): The recommendation for role models is specifically designed to provide an "alternate vision" to adolescent motherhood. Role models help girls develop aspirations for school completion and income-generating jobs, countering the community norm that motherhood is the only viable path.

The Role of Schools and Organizations Similarities: Both studies recommend multisectoral collaboration. They agree that schools are strategic platforms for disseminating information.
Differences in Implementation:In Ethiopia, the policy recommendation is to integrate SRH into the formal curriculum as a subject (e.g., Grade 1 HIV-related curriculum).In Malawi, while school is valued, there is a heavier emphasis on out-of-school approaches, such as girl groups led by these "outside experts" to reach those who have already dropped out or to supplement inconsistent school-based education.

---

[1] Nash et al. Reproductive Health (2019) 16:8 https://doi.org/10.1186/s12978-018-0661-x

Feature	Malawi Study (2019)	Ethiopia Study (Manuscript)
**Study Design**	Formative research using semi-structured **individual interviews**.	Qualitative study using **Focus Group Discussions (FGDs)**.
**Participant Groups**	Triangulated: Adolescent girls, mothers/female guardians (M/FGs), and initiation counselors.	Single perspective: Parents and guardians of youth.
**Sample Size**	**N=40** (18 girls, 12 M/FGs, 10 counselors).	**N=12** (6 males, 6 females).
**Location**	15 villages in Mulanje District (rural, southern).	Gurage Zone, Southern Ethiopia.
**Analysis Method**	Thematic coding using Dedoose 7.5.	Thematic analysis using Braun and Clarke framework.
