# Supplementary material for: Parents’ and guardians’ perceptions of sexual and reproductive health communication with youth: A qualitative study in Gurage Zone, Southern Ethiopia
Source: PLOS Glob Public Health. 2026 Jul 13;6(7):e0006825. doi: 10.1371/journal.pgph.0006825 (PMC13362135; doi:10.1371/journal.pgph.0006825)
Supplement: Attachment — Submitted filename: Commentaires_2026 04 21.docx [file pgph.0006825.s003.docx]

This manuscript addresses a relevant and important topic. The study contributes to understanding parental perceptions of sexual and reproductive health (SRH) communication in a culturally specific context. However, several methodological and reporting aspects require clarification or strengthening to enhance transparency and alignment with qualitative research standards.

**General Comment**

- It is unclear whether the authors used a qualitative reporting guideline such as COREQ. If so, this should be explicitly stated. If not, the authors are encouraged to align their reporting with a qualitative reporting guideline to improve methodological transparency.

**Methods and Materials**

**Study Design**

- The manuscript states that *“The study was conducted in Gurage Zone in Southern Ethiopia.”* The rationale for selecting this specific study site is not provided. If the study is part of a broader project conducted in this area, please specify.
- The authors should justify the choice of focus group discussions over individual interviews or other qualitative data collection methods. Clarifying why FGDs were deemed most appropriate for exploring parental perceptions would strengthen the methodological justification.
- The statement *“To minimize bias, researchers maintained reflexive awareness of their own perspectives…”* requires further elaboration. How was reflexivity operationalized in practice? For example, did the team use reflexive journals, debriefing sessions, or positionality statements?

**Participant Recruitment**

- The inclusion criterion *“residing in the study area”* lacks operational definition. The authors do not indicate whether a minimum duration of residence was required (e.g., 6 months, 1 year), nor whether temporary or newly arrived residents were excluded. In qualitative research exploring culturally embedded perceptions, the length and stability of residence can significantly influence participants’ familiarity with local norms and communication practices.
- The criterion *“parent or guardian of youth”* is also insufficiently defined. The authors do not specify whether participants had to be adults, nor whether adolescent parents (e.g., a 15‑year‑old mother) were eligible. Please, clarify the targeted age range for parental participants.

**Data Collection**

- The manuscript states that data were collected using a semi‑structured interview guide, but it is unclear how this guide was developed. Was a conceptual or theoretical framework used? Who was involved in designing and validating the guide? Please indicate whether the interview guide underwent pretesting or piloting prior to data collection.

**Data Analysis**

- Please specify the exact number of researchers involved in the analysis.
- It would also be helpful to indicate whether differences in coding or interpretation emerged, and if so, how these were resolved (e.g., consensus meetings, third‑party adjudication).
- Clarify whether the research team brought diverse disciplinary or experiential perspectives.
- A critical methodological element is missing from this section: you do not discuss how data saturation was assessed or achieved. Saturation is a central criterion in qualitative research. The manuscript does not indicate whether saturation was monitored during data collection, how the research team determined that no new themes were emerging, or whether the number of participants and FGDs was sufficient to reach thematic saturation.

**Results**

**Participants’ Demographic Information**

- The manuscript indicates that *“FGD involved 12 participants (6 females) aged between 40 and 68 years.”* However, it is unclear how many focus group discussions were conducted in total, and how many participants took part in each group.

**Theme Presentation**

- Under *Theme 2: Parental attitudes and beliefs on SRH communication*, the introductory paragraph states that four sub‑categories were identified. However, only one sub‑theme appears to be presented in the text. Please verify whether the remaining sub‑themes were omitted inadvertently.
- More generally, ensure that the introductory paragraph for each theme accurately reflects the sub‑themes that follow, and that all themes and sub‑themes listed in Table 2 are fully presented in the Results section.

**Discussion**

- The discussion is generally clear but remains overly descriptive. It largely restates the results rather than offering deeper analytical interpretation or critical engagement with the literature.
- The authors cite previous studies but do not articulate how their findings confirm, extend, or challenge existing evidence.
- The authors may also consider comparing their findings with those of our study entitled *“Parental Communication on Sexual and Reproductive Health in Côte d’Ivoire: An Interpretive Description Study in the Haut‑Sassandra Region.”*
- The study identifies several strategies and enablers for effective communication. These should be discussed in greater depth, with concrete recommendations for practice, policy, and community interventions.
- The implications for practice remain vague. The discussion should more clearly articulate how the findings inform specific interventions, which stakeholders should be involved, and how these strategies could be operationalized.

**Limitations**

- The manuscript does not discuss key methodological limitations. The authors should address: the exclusion of adolescents’ and youths’ perspectives; the implications (bias) of conducting interviews in the local language and translating transcripts into English; potential translation‑related loss of meaning; the small sample size; the absence of information on data saturation; possible social desirability bias during FGDs; etc.
