# Supplementary material for: Parents’ and guardians’ perceptions of sexual and reproductive health communication with youth: A qualitative study in Gurage Zone, Southern Ethiopia
Source: PLOS Glob Public Health. 2026 Jul 13;6(7):e0006825. doi: 10.1371/journal.pgph.0006825 (PMC13362135; doi:10.1371/journal.pgph.0006825)
Supplement: Attachment — Submitted filename: RESPON~1.DOC [file pgph.0006825.s005.DOC]

This manuscript addresses a relevant and important topic. The study contributes to understanding parental perceptions of sexual and reproductive health (SRH) communication in a culturally specific context. However, several methodological and reporting aspects require clarification or strengthening to enhance transparency and alignment with qualitative research standards.

- *We sincerely thank the academic editor and all three reviewers for the careful assessment of the manuscript and for the constructive comments provided. We are encouraged that the manuscript was considered to have merit and that the topic was recognized as relevant to PLOS Global Public Health. we have revised the manuscript substantially, particularly the methodology, results, discussion, conclusion, data availability statement and limitations sections.*

*We have addressed each comment point-by-point below. In the revised manuscript, we have strengthened methodological transparency, clarified the number and composition of focus group discussions, expanded the study setting, explained the rationale for using FGDs, described reflexivity and saturation, improved the presentation of themes and sub-themes, added more quotations to support the findings, deepened the discussion, clarified practical and policy implications and revised the limitations section.*

**General Comment**

- It is unclear whether the authors used a qualitative reporting guideline such as COREQ. If so, this should be explicitly stated. If not, the authors are encouraged to align their reporting with a qualitative reporting guideline to improve methodological transparency.
- *Thank you for this important recommendation. We have revised the manuscript to state that reporting was guided by the Consolidated Criteria for Reporting Qualitative Research (COREQ). We also revised the Methods section to better align with COREQ domains including research team characteristics, study design, participant selection, data collection, analysis, reflexivity and reporting.*

**Methods and Materials**

**Study Design**

- The manuscript states that *“The study was conducted in Gurage Zone in Southern Ethiopia.”* The rationale for selecting this specific study site is not provided. If the study is part of a broader project conducted in this area, please specify.
- *Thank you for this comment. We agree that the rationale for selecting Gurage Zone required clarification. The manuscript has been revised to explain that Gurage Zone was selected because the study formed part of a broader doctoral research project involving youth, healthcare workers and examining parent–youth SRH communication in Southern Ethiopia. The area was also relevant because it is characterized by strong cultural and religious traditions that may shape family communication on sexuality and reproductive health.*
- The authors should justify the choice of focus group discussions over individual interviews or other qualitative data collection methods. Clarifying why FGDs were deemed most appropriate for exploring parental perceptions would strengthen the methodological justification.
- *We appreciate this observation. We have revised the study design section to justify the use of FGDs. FGDs were considered appropriate because the study aimed to explore shared community norms, collective parental perceptions, social expectations and culturally embedded beliefs regarding SRH communication. Since SRH communication is shaped by family, community, religious and cultural norms, group interaction allowed participants to reflect on, compare and discuss shared experiences*.
- The statement *“To minimize bias, researchers maintained reflexive awareness of their own perspectives…”* requires further elaboration. How was reflexivity operationalized in practice? For example, did the team use reflexive journals, debriefing sessions, or positionality statements?
- *Thank you for this helpful comment. We agree that the original statement was too brief. We have expanded the methods section to clarify how reflexivity was practiced. The research team used post-discussion debriefings, reflective notes and team discussions to examine how researchers’ backgrounds, assumptions and professional experience could influence data collection and interpretation.*

**Participant Recruitment**

- The inclusion criterion *“residing in the study area”* lacks operational definition. The authors do not indicate whether a minimum duration of residence was required (e.g., 6 months, 1 year), nor whether temporary or newly arrived residents were excluded. In qualitative research exploring culturally embedded perceptions, the length and stability of residence can significantly influence participants’ familiarity with local norms and communication practices.
- *We agree with the reviewer. We have revised the participant recruitment section to define residence more clearly. Participants were eligible if they had lived in the study area for at least six months and were familiar with the local social and cultural context.*
- The criterion *“parent or guardian of youth”* is also insufficiently defined. The authors do not specify whether participants had to be adults, nor whether adolescent parents (e.g., a 15‑year‑old mother) were eligible. Please, clarify the targeted age range for parental participants.
- Thank you. We have clarified that participants were adult parents or guardians responsible for youth and who had parental or caregiving responsibility for youth.

**Data Collection**

- The manuscript states that data were collected using a semi‑structured interview guide, but it is unclear how this guide was developed. Was a conceptual or theoretical framework used? Who was involved in designing and validating the guide? Please indicate whether the interview guide underwent pretesting or piloting prior to data collection.
- We appreciate this comment. We have revised the data collection section to explain that the guide was developed based on the study objectives, literature on parent–youth SRH communication and the broader doctoral research focus. It was reviewed by the research supervisors and refined before data collection. We also clarified that the guide was pretested/piloted to assess clarity, sensitivity and flow.

**Data Analysis**

- Please specify the exact number of researchers involved in the analysis.
- Thank you. We have revised the data analysis section to specify the researchers were involved in reviewing and interpreting the data.
- It would also be helpful to indicate whether differences in coding or interpretation emerged, and if so, how these were resolved (e.g., consensus meetings, third‑party adjudication).
- We agree that this required clarification. We have revised the manuscript to explain that disagreements were discussed through consensus meetings.
- Clarify whether the research team brought diverse disciplinary or experiential perspectives.
- Thank you for this suggestion. We have revised the methods section to clarify that the team brought experience in public health, qualitative research, health studies and SRH-related research. This diversity strengthened interpretation and reflexive discussion.
- A critical methodological element is missing from this section: you do not discuss how data saturation was assessed or achieved. Saturation is a central criterion in qualitative research. The manuscript does not indicate whether saturation was monitored during data collection, how the research team determined that no new themes were emerging, or whether the number of participants and FGDs was sufficient to reach thematic saturation.
- We appreciate this important methodological comment. We have revised the data analysis and trustworthiness section to clarify how saturation was monitored. During data collection and analysis, the research team reviewed emerging codes and themes after each FGD. Saturation was considered reached when additional discussions did not generate substantially new themes but reinforced already identified categories such as openness, cultural barriers, gendered communication, parental knowledge gaps, and community-based strategies.

**Results**

**Participants’ Demographic Information**

- The manuscript indicates that *“FGD involved 12 participants (6 females) aged between 40 and 68 years.”* However, it is unclear how many focus group discussions were conducted in total, and how many participants took part in each group.
- Thank you. We have clarified this in the results section. The total participant number was 12. We have revised the manuscript to state that two FGDs were conducted, each with six participants.

**Theme Presentation**

- Under *Theme 2: Parental attitudes and beliefs on SRH communication*, the introductory paragraph states that four sub‑categories were identified. However, only one sub‑theme appears to be presented in the text. Please verify whether the remaining sub‑themes were omitted inadvertently.
- We fully agree with the reviewer. This was an important omission. We have revised the results section to ensure that all four sub-categories under Theme 2 are fully presented: perceived importance of SRH communication, parental attitudes, gendered perspectives and intergenerational learning and role modelling. These sub-themes were available in the original mother research result but were not sufficiently transferred into the manuscript.
- More generally, ensure that the introductory paragraph for each theme accurately reflects the sub‑themes that follow, and that all themes and sub‑themes listed in Table 2 are fully presented in the Results section.
- Thank you. We have carefully checked and revised the results section to ensure consistency between Table 2 and the narrative presentation.

**Discussion**

- The discussion is generally clear but remains overly descriptive. It largely restates the results rather than offering deeper analytical interpretation or critical engagement with the literature.
- We appreciate this valuable critique. We have substantially revised the 7discussion section to move beyond description and provide deeper interpretation. The revised discussion now explains how the findings reflect the tension between parental recognition of SRH communication and cultural discomfort in practicing it. It also analyzes how gender norms, parental authority, limited literacy, economic pressures and intergenerational differences shape communication patterns.
- The authors cite previous studies but do not articulate how their findings confirm, extend, or challenge existing evidence.
- Thank you. We revised the discussion to make clearer links between our findings and existing literature. The revised manuscript explains that our findings confirm previous evidence that cultural taboos, gender norms, and parental discomfort limit SRH communication. The study extends existing evidence by showing how parents in Gurage Zone also identify practical solutions including safe spaces, early initiation, peer education, media guidance, school involvement, religious institutions, and community-level engagement.
- The authors may also consider comparing their findings with those of our study entitled *“Parental Communication on Sexual and Reproductive Health in Côte d’Ivoire: An Interpretive Description Study in the Haut‑Sassandra Region.”*
- Thank you for recommending this relevant study. We have incorporated a comparative discussion with the Côte d’Ivoire study. The comparison strengthens the regional interpretation of the findings and shows that silence, discomfort, cultural norms and gendered patterns are not unique to Ethiopia but reflect broader African sociocultural dynamics. At the same time, our study contributes setting-specific evidence from Gurage Zone.
- The study identifies several strategies and enablers for effective communication. These should be discussed in greater depth, with concrete recommendations for practice, policy, and community interventions.
- We agree. The discussion has been expanded to provide more concrete interpretation and recommendations. We now discuss parental communication training, community dialogue, school-based SRH education, engagement of religious leaders, peer education, youth-friendly health services and media literacy.
- The implications for practice remain vague. The discussion should more clearly articulate how the findings inform specific interventions, which stakeholders should be involved, and how these strategies could be operationalized.
- Thank you. We have revised the implications section to clearly identify stakeholders and practical actions.

**Limitations**

- The manuscript does not discuss key methodological limitations. The authors should address: the exclusion of adolescents’ and youths’ perspectives; the implications (bias) of conducting interviews in the local language and translating transcripts into English; potential translation‑related loss of meaning; the small sample size; the absence of information on data saturation; possible social desirability bias during FGDs; etc.
- We agree. We have added a more comprehensive limitations section addressing these issues.
