# Supplementary material for: Parents’ and guardians’ perceptions of sexual and reproductive health communication with youth: A qualitative study in Gurage Zone, Southern Ethiopia
Source: PLOS Glob Public Health. 2026 Jul 13;6(7):e0006825. doi: 10.1371/journal.pgph.0006825 (PMC13362135; doi:10.1371/journal.pgph.0006825)
Supplement: Peer review history

# Author comment: Author response to Decision Letter 0

**DOI:** 10.1371/journal.pgph.0006825.r001

26 Mar 2026

Attachment Submitted filename: Justication for the comment.pdf

# Peer review: Decision Letter 0

**DOI:** 10.1371/journal.pgph.0006825.r002

**Authors:** Dumile Gumede

22 Apr 2026

PGPH-D-26-01021

Parents’ and Guardians’ Perceptions of Sexual and Reproductive Health Communication with Youth: A Qualitative Study in Gurage Zone, Southern Ethiopia

PLOS Global Public Health

Dear Dr. Nida,

Thank you for submitting your manuscript to PLOS Global Public Health. After careful consideration, we feel that it has merit but does not fully meet PLOS Global Public Health’s publication criteria as it currently stands. Therefore, we invite you to submit a revised version of the manuscript that addresses the points raised during the review process.

After careful consideration of the reviewers’ reports, it is clear that all three reviewers have identified significant gaps in the paper, particularly within the methodology, discussion, and conclusion sections. I concur with these assessments.

Please submit your revised manuscript by Jun 06 2026 11:59PM. If you will need more time than this to complete your revisions, please reply to this message or contact the journal office at globalpubhealth@plos.org. When you're ready to submit your revision, log on to https://www.editorialmanager.com/pgph/ and select the 'Submissions Needing Revision' folder to locate your manuscript file.

Please include the following items when submitting your revised manuscript:

- A letter that responds to each point raised by the editor and reviewer(s). You should upload this letter as a separate file labeled 'Response to Reviewers'.
- A marked-up copy of your manuscript that highlights changes made to the original version. You should upload this as a separate file labeled 'Revised Manuscript with Track Changes'.
- An unmarked version of your revised paper without tracked changes. You should upload this as a separate file labeled 'Manuscript'.

Guidelines for resubmitting your figure files are available below the reviewer comments at the end of this letter.

As the corresponding author, your ORCID iD is verified in the submission system and will appear in the published article. PLOS supports the use of ORCID, and we encourage all coauthors to register for an ORCID iD and use it as well. Please encourage your coauthors to verify their ORCID iD within the submission system before final acceptance, as unverified ORCID iDs will not appear in the published article. *Only*  the individual author can complete the verification step; PLOS staff *cannot*  verify ORCID iDs on behalf of authors.

We look forward to receiving your revised manuscript.

Kind regards,

Dumile Gumede, PhD (Health Promotion)

Academic Editor

PLOS Global Public Health

**Journal Requirements:**

1. Please include a complete copy of PLOS’ questionnaire on inclusivity in global research in your revised manuscript. Our policy for research in this area aims to improve transparency in the reporting of research performed outside of researchers’ own country or community. The policy applies to researchers who have travelled to a different country to conduct research, research with Indigenous populations or their lands, and research on cultural artefacts. The questionnaire can also be requested at the journal’s discretion for any other submissions, even if these conditions are not met. Please find more information on the policy and a link to download a blank copy of the questionnaire here: https://journals.plos.org/globalpublichealth/s/best-practices-in-research-reporting. Please upload a completed version of your questionnaire as Supporting Information when you resubmit your manuscript.

2. In the online submission form, you indicated that “The datasets generated during this study are available from the corresponding author upon reasonable request.”.

All PLOS journals now require all data underlying the findings described in their manuscript to be freely available to other researchers, either

1. In a public repository,

2. Within the manuscript itself, or

3. Uploaded as supplementary information.

This policy applies to all data except where public deposition would breach compliance with the protocol approved by your research ethics board. If your data cannot be made publicly available for ethical or legal reasons (e.g., public availability would compromise patient privacy), please explain your reasons by return email and your exemption request will be escalated to the editor for approval. Your exemption request will be handled independently and will not hold up the peer review process, but will need to be resolved should your manuscript be accepted for publication. One of the Editorial team will then be in touch if there are any issues.

3. Please ensure that your Ethics Statement is available in its entirety at the beginning of your Methods section, under a subheading 'Ethics Statement'.

If the reviewer comments include a recommendation to cite specific previously published works, please review and evaluate these publications to determine whether they are relevant and should be cited. There is no requirement to cite these works unless the editor has indicated otherwise.

**Additional Editor Comments (if provided):**

[Note: HTML markup is below. Please do not edit.]

Reviewers' comments:

Reviewer's Responses to Questions

**Comments to the Author**

1. Does this manuscript meet PLOS Global Public Health’s publication criteria? Is the manuscript technically sound, and do the data support the conclusions? The manuscript must describe methodologically and ethically rigorous research with conclusions that are appropriately drawn based on the data presented.

Reviewer #1: Partly

Reviewer #2: No

Reviewer #3: Yes

**********

2. Has the statistical analysis been performed appropriately and rigorously?

Reviewer #1: No

Reviewer #2: N/A

Reviewer #3: N/A

**********

3. Have the authors made all data underlying the findings in their manuscript fully available (please refer to the Data Availability Statement at the start of the manuscript PDF file)?

The PLOS Data policy requires authors to make all data underlying the findings described in their manuscript fully available without restriction, with rare exception. The data should be provided as part of the manuscript or its supporting information, or deposited to a public repository. For example, in addition to summary statistics, the data points behind means, medians and variance measures should be available. If there are restrictions on publicly sharing data—e.g. participant privacy or use of data from a third party—those must be specified.

Reviewer #1: Yes

Reviewer #2: No

Reviewer #3: No

**********

4. Is the manuscript presented in an intelligible fashion and written in standard English?

PLOS Global Public Health does not copyedit accepted manuscripts, so the language in submitted articles must be clear, correct, and unambiguous. Any typographical or grammatical errors should be corrected at revision, so please note any specific errors here.

Reviewer #1: Yes

Reviewer #2: Yes

Reviewer #3: Yes

**********

5. Review Comments to the Author

Please use the space provided to explain your answers to the questions above. You may also include additional comments for the author, including concerns about dual publication, research ethics, or publication ethics. (Please upload your review as an attachment if it exceeds 20,000 characters)

**Reviewer #1:**  Based on the provided manuscript, the Ethiopian study utilized a qualitative approach focused on a specific geographic and demographic subset. Feedback based on the review are as follows;

1. The sample size and its implications for the research findings:

Overview: The study consisted of 12 participants. The group was balanced by gender, including 6 females and 6 males. The participants were aged between 40 and 68 years and all participants were residents of the Gurage Zone in Southern Ethiopia. The study employed purposive sampling. This non-probability sampling method was chosen to ensure representation from parents and guardians with diverse backgrounds, including variations in education (ranging from uneducated to degree holders) and employment (housewives, merchants, civil servants, and farmers).

Depth of Exploration: This is compromised by the small sample size (N=12), the researchers utilized Focus Group Discussions (FGDs) lasting 60–90 minutes to capture rich, descriptive data. This allowed for the emergence of nuanced themes regarding sociocultural taboos and "secrecy," which might be harder to quantify in a larger survey-based study. In qualitative research, the goal is often data saturation—the point where no new information or themes emerge from the data. The researchers identified four major themes (perceptions, attitudes/beliefs, barriers, and strategies) from this group, suggesting that despite the small number, the participants provided sufficient diversity in thought to establish a comprehensive framework for the study area

Limited Generalizability: The small sample size and specific focus on the Gurage Zone mean the findings cannot be statistically generalized to the entire Ethiopian population

Impact on Sub-group Analysis: The small size (N=12) limits the ability to perform detailed comparisons between different demographic sub-groups within the sample. For example, while the study notes that all four widowed participants were women and that women generally had lower education levels than men, the sample is too small to definitively correlate these specific traits with unique SRH communication styles.

Mitigation of Bias: To counter the potential limitations of a small sample, the study implemented trustworthiness strategies, such as involving multiple researchers to review transcripts (credibility) and grounding interpretations strictly in the participants' direct narratives (confirmability)

2. Policy recommendations: Based on the study conducted in the Gurage Zone, Southern Ethiopia, the researchers provided several key recommendations aimed at overcoming cultural barriers and structural challenges to improve youth sexual and reproductive health (YSRH) to focus on broader systemic changes to support family-based SRH communication

Suggestions for structuring the policy recommendations as follows, despite the limited confidence that this research has produced adequate evidence to support such recommendations.

a. Multisectoral Collaboration: The study advocates for strong collaboration between families, schools, health institutions, government ministries, and NGOs to advance SRH initiatives.

b. Early Education Policies: Policies should support the initiation of SRH education during early adolescence (starting as early as age 9 or 10) to normalize the topic before youth are exposed to misinformation.

c. Supportive Educational Curricula: Formal adoption and expansion of SRH and HIV-related curricula in schools, starting from early grades (e.g., Grade 1), to build a foundational understanding of risks and impacts.

d. Addressing Economic and Structural Barriers: Policymakers need to recognize that competing socioeconomic responsibilities and economic pressures limit parental engagement, suggesting a need for broader support systems for families.

e. Integration of Services: Enhancing the reach of youth-friendly health services by ensuring they are culturally appropriate and supported by community awareness programs.

Comparison to similar Malawi Study (Nash et al. Reproductive Health(2019) 16:8 https://doi.org/10.1186/s12978-018-0661-x):

The comparison between the Ethiopian and Malawian recommendations reveals a shift in who is perceived as the most effective messenger for sexual and reproductive health (SRH) information. While the Ethiopia study focuses on strengthening existing community and family structures, the Malawi study emphasizes the need for external figures to break through local cultural constraints. The Malawi study provides alternative vision to addressing the communication gap.

Ethiopia Study (Guidance & Risk Management): The focus is on risk reduction and healthy decision-making. Parents and stakeholders are encouraged to create "safe spaces" to help youth avoid "bad friends," "harmful content" on smartphones, and "immorality".

Malawi Study (Aspiration & Future Paths): The recommendation for role models is specifically designed to provide an "alternate vision" to adolescent motherhood. Role models help girls develop aspirations for school completion and income-generating jobs, countering the community norm that motherhood is the only viable path

I recommend that the authors revisit the sample size and consider a larger sample and wider coverage. I also recommend that the authors revisit the sample representation and provide alternative vision by including role models and other community agents.

**Reviewer #2:** General Comments

1. Original data not deposited in any repositories, but the authors said it could be provided on reasonable request

2. The details provided under the methodology section are not sufficient enough to support reproducibility

Abstract

• There is no mention of the total number of FGDs done

Background

• Provide reference for last sentence on lines 70/71

• Provide more empirical evidence to support your problem statement

Material and Methods

• This section needs a major review because it is too vague and evasive. Just 1 FGD was reported, and this is not enough to provide results that can be generalized.

o Provide more context to enable the audience to understand the study setting better

• Geographical map

• Population size

• Prevailing sociodemographic characteristics

o Data collection: how many FGDs were conducted? What is the distribution of the participants? Were they segregated according to sex, or were the participants mixed? Did you have notetakers sit in the interviews?

o Implement lines 157 to 159 in this section to enable readers to actually assess the applicability of the findings to other settings

Results

• The results presented are not enough to support the conclusion

• In instances where the authors reported that most participants agreed on a theme, there should be more than 1 response (quoted comments) to support that claim. For example, lines 209 – 211; 231 – 233.

• The themes are not well presented. What is the difference between the first (perceptions) and second (attitudes and beliefs) themes reported? The sub-themes presented under these themes were disjointed.

**Reviewer #3:**  This manuscript addresses a relevant and important topic. The study contributes to understanding parental perceptions of sexual and reproductive health (SRH) communication in a culturally specific context. However, several methodological and reporting aspects require clarification or strengthening to enhance transparency and alignment with qualitative research standards.

General Comment

• It is unclear whether the authors used a qualitative reporting guideline such as COREQ. If so, this should be explicitly stated. If not, the authors are encouraged to align their reporting with a qualitative reporting guideline to improve methodological transparency.

Methods and Materials

Study Design

• The manuscript states that “The study was conducted in Gurage Zone in Southern Ethiopia.” The rationale for selecting this specific study site is not provided. If the study is part of a broader project conducted in this area, please specify.

• The authors should justify the choice of focus group discussions over individual interviews or other qualitative data collection methods. Clarifying why FGDs were deemed most appropriate for exploring parental perceptions would strengthen the methodological justification.

• The statement “To minimize bias, researchers maintained reflexive awareness of their own perspectives…” requires further elaboration. How was reflexivity operationalized in practice? For example, did the team use reflexive journals, debriefing sessions, or positionality statements?

Participant Recruitment

• The inclusion criterion “residing in the study area” lacks operational definition. The authors do not indicate whether a minimum duration of residence was required (e.g., 6 months, 1 year), nor whether temporary or newly arrived residents were excluded. In qualitative research exploring culturally embedded perceptions, the length and stability of residence can significantly influence participants’ familiarity with local norms and communication practices.

• The criterion “parent or guardian of youth” is also insufficiently defined. The authors do not specify whether participants had to be adults, nor whether adolescent parents (e.g., a 15 year old mother) were eligible. Please, clarify the targeted age range for parental participants.

Data Collection

• The manuscript states that data were collected using a semi structured interview guide, but it is unclear how this guide was developed. Was a conceptual or theoretical framework used? Who was involved in designing and validating the guide? Please indicate whether the interview guide underwent pretesting or piloting prior to data collection.

Data Analysis

• Please specify the exact number of researchers involved in the analysis.

• It would also be helpful to indicate whether differences in coding or interpretation emerged, and if so, how these were resolved (e.g., consensus meetings, third party adjudication).

• Clarify whether the research team brought diverse disciplinary or experiential perspectives.

• A critical methodological element is missing from this section: you do not discuss how data saturation was assessed or achieved. Saturation is a central criterion in qualitative research. The manuscript does not indicate whether saturation was monitored during data collection, how the research team determined that no new themes were emerging, or whether the number of participants and FGDs was sufficient to reach thematic saturation.

Results

Participants’ Demographic Information

• The manuscript indicates that “FGD involved 12 participants (6 females) aged between 40 and 68 years.” However, it is unclear how many focus group discussions were conducted in total, and how many participants took part in each group.

Theme Presentation

• Under Theme 2: Parental attitudes and beliefs on SRH communication, the introductory paragraph states that four sub categories were identified. However, only one sub theme appears to be presented in the text. Please verify whether the remaining sub themes were omitted inadvertently.

• More generally, ensure that the introductory paragraph for each theme accurately reflects the sub themes that follow, and that all themes and sub themes listed in Table 2 are fully presented in the Results section.

Discussion

• The discussion is generally clear but remains overly descriptive. It largely restates the results rather than offering deeper analytical interpretation or critical engagement with the literature.

• The authors cite previous studies but do not articulate how their findings confirm, extend, or challenge existing evidence.

• The authors may also consider comparing their findings with those of our study entitled “Parental Communication on Sexual and Reproductive Health in Côte d’Ivoire: An Interpretive Description Study in the Haut Sassandra Region.”

• The study identifies several strategies and enablers for effective communication. These should be discussed in greater depth, with concrete recommendations for practice, policy, and community interventions.

• The implications for practice remain vague. The discussion should more clearly articulate how the findings inform specific interventions, which stakeholders should be involved, and how these strategies could be operationalized.

Limitations

• The manuscript does not discuss key methodological limitations. The authors should address: the non inclusion of adolescents’ and youths’ perspectives; the implications (bias) of conducting interviews in the local language and translating transcripts into English; potential translation related loss of meaning; the small sample size; the absence of information on data saturation; possible social desirability bias during FGDs; etc.

**********

6. PLOS authors have the option to publish the peer review history of their article (what does this mean?). If published, this will include your full peer review and any attached files.

**Do you want your identity to be public for this peer review?**  If you choose “no”, your identity will remain anonymous but your review may still be made public.

For information about this choice, including consent withdrawal, please see our Privacy Policy.

Reviewer #1: **Yes:** Olanike Adedeji

Reviewer #2: No

Reviewer #3: **Yes:** Titilayo Tatiana Agbadje

**********

[NOTE: If reviewer comments were submitted as an attachment file, they will be attached to this email and accessible via the submission site. Please log into your account, locate the manuscript record, and check for the action link "View Attachments". If this link does not appear, there are no attachment files.]

**Figure Resubmissions:**

While revising your submission, we strongly recommend that you use PLOS’s NAAS tool (https://ngplosjournals.pagemajik.ai/artanalysis) to test your figure files. NAAS can convert your figure files to the TIFF file type and meet basic requirements (such as print size, resolution), or provide you with a report on issues that do not meet our requirements and that NAAS cannot fix.

After uploading your figures to PLOS’s NAAS tool - https://ngplosjournals.pagemajik.ai/artanalysis, NAAS will process the files provided and display the results in the "Uploaded Files" section of the page as the processing is complete. If the uploaded figures meet our requirements (or NAAS is able to fix the files to meet our requirements), the figure will be marked as "fixed" above. If NAAS is unable to fix the files, a red "failed" label will appear above. When NAAS has confirmed that the figure files meet our requirements, please download the file via the download option, and include these NAAS processed figure files when submitting your revised manuscript.

Attachment Submitted filename: Feedback_Reviewer.pdf

Attachment Submitted filename: Commentaires_2026 04 21.docx

# Author comment: Author response to Decision Letter 1

**DOI:** 10.1371/journal.pgph.0006825.r003

22 May 2026

Attachment Submitted filename: RESPON~1.DOC

# Peer review: Decision Letter 1

**DOI:** 10.1371/journal.pgph.0006825.r004

**Authors:** Dumile Gumede, Dumile Gumede

18 Jun 2026

Parents’ and Guardians’ Perceptions of Sexual and Reproductive Health Communication with Youth: A Qualitative Study in Gurage Zone, Southern Ethiopia

PGPH-D-26-01021R1

Dear Mr Nida,

We are pleased to inform you that your manuscript 'Parents’ and Guardians’ Perceptions of Sexual and Reproductive Health Communication with Youth: A Qualitative Study in Gurage Zone, Southern Ethiopia' has been provisionally accepted for publication in PLOS Global Public Health.

Before your manuscript can be formally accepted you will need to complete some formatting changes, which you will receive in a follow up email. A member of our team will be in touch with a set of requests.

Please note that your manuscript will not be scheduled for publication until you have made the required changes, so a swift response is appreciated.

IMPORTANT: The editorial review process is now complete. PLOS will only permit corrections to spelling, formatting or significant scientific errors from this point onwards. Requests for major changes, or any which affect the scientific understanding of your work, will cause delays to the publication date of your manuscript.

If your institution or institutions have a press office, please notify them about your upcoming paper to help maximize its impact. If they'll be preparing press materials, please inform our press team as soon as possible -- no later than 48 hours after receiving the formal acceptance. Your manuscript will remain under strict press embargo until 2 pm Eastern Time on the date of publication. For more information, please contact globalpubhealth@plos.org.

Thank you again for supporting Open Access publishing; we are looking forward to publishing your work in PLOS Global Public Health.

Best regards,

Dumile Gumede, PhD (Health Promotion)

Academic Editor

PLOS Global Public Health

***********************************************************

Reviewer Comments (if any, and for reference):

Reviewer's Responses to Questions

**Comments to the Author**

1. If the authors have adequately addressed your comments raised in a previous round of review and you feel that this manuscript is now acceptable for publication, you may indicate that here to bypass the “Comments to the Author” section, enter your conflict of interest statement in the “Confidential to Editor” section, and submit your "Accept" recommendation.

Reviewer #1: All comments have been addressed

Reviewer #2: All comments have been addressed

**********

2. Does this manuscript meet PLOS Global Public Health’s publication criteria? Is the manuscript technically sound, and do the data support the conclusions? The manuscript must describe methodologically and ethically rigorous research with conclusions that are appropriately drawn based on the data presented.

Reviewer #1: Yes

Reviewer #2: Yes

**********

3. Has the statistical analysis been performed appropriately and rigorously?

Reviewer #1: Yes

Reviewer #2: N/A

**********

4. Have the authors made all data underlying the findings in their manuscript fully available (please refer to the Data Availability Statement at the start of the manuscript PDF file)?

The PLOS Data policy requires authors to make all data underlying the findings described in their manuscript fully available without restriction, with rare exception. The data should be provided as part of the manuscript or its supporting information, or deposited to a public repository. For example, in addition to summary statistics, the data points behind means, medians and variance measures should be available. If there are restrictions on publicly sharing data—e.g. participant privacy or use of data from a third party—those must be specified.

Reviewer #1: Yes

Reviewer #2: No

**********

5. Is the manuscript presented in an intelligible fashion and written in standard English?

PLOS Global Public Health does not copyedit accepted manuscripts, so the language in submitted articles must be clear, correct, and unambiguous. Any typographical or grammatical errors should be corrected at revision, so please note any specific errors here.

Reviewer #1: Yes

Reviewer #2: Yes

**********

6. Review Comments to the Author

Please use the space provided to explain your answers to the questions above. You may also include additional comments for the author, including concerns about dual publication, research ethics, or publication ethics. (Please upload your review as an attachment if it exceeds 20,000 characters)

Reviewer #1: No further review recommendations

Reviewer #2: Though the authors did not respond provide any direct response to my previous comments, I can attest to a significant improvement in the updated manuscript.

Irecommend that the authors correct the few typographical errors before final submission.

**********

7. PLOS authors have the option to publish the peer review history of their article (what does this mean?). If published, this will include your full peer review and any attached files.

**Do you want your identity to be public for this peer review?**  If you choose “no”, your identity will remain anonymous but your review may still be made public.

For information about this choice, including consent withdrawal, please see our Privacy Policy.

Reviewer #1: **Yes:** Olanike Adedeji

Reviewer #2: No

**********
